# Ethnic Differences in Attitudes and Preventive Behaviors Related to Alzheimer’s Disease in the Israeli Survey of Aging

**DOI:** 10.3390/ijerph19159705

**Published:** 2022-08-06

**Authors:** Efrat Neter, Svetlana Chachashvili-Bolotin

**Affiliations:** 1 Department of Behavioral Sciences, Ruppin Academic Center, Emeq Hefer 4025000, Israel; 2 Institute for Immigration & Social Integration, Ruppin Academic Center, Emeq Hefer 4025000, Israel

**Keywords:** Alzheimer’s disease, attitudes, ethnicity, health behaviors, immigration, internet use

## Abstract

Objectives: To examine ethnic differences in attitudes and preventive behaviors related to Alzheimer’s Disease (AD) in Israel. Methods: A household representative sample included 1198 older adults (*M* age = 70.78, SD = 9.64) who participated in the Israeli branch of the Survey of Health, Aging, and Retirement in Europe (SHARE-Israel), collected during 2015 and 2017. Descriptions of the groups (long term Israeli Jews (LTIJ), immigrants from the Former Soviet Union (FSU) and Palestinian Citizens of Israel (PCI)) were computed, and hierarchical regressions tested whether group differences were maintained after controlling for demographic, human and economic resources, Internet use, and AD familiarity. Results: Attitudes towards AD were the most negative among FSU and more accepting among PCI while AD-related preventive behaviors were highest among FSU, lowest among PCI, with LTIJ between them. After including demographic, human and economic resources, and familiarity with AD, differences in AD-preventive behaviors significantly decreased. In contrast, differences in attitudes among the groups remained stable even after other variables were accounted for, so that PCI were the most accepting and FSU manifested greatest avoidance of contact with persons with AD. Conclusions: The findings provide directions for culturally sensitive psycho-educational and other interventions for both the public and healthcare providers.

## 1. Introduction

Alzheimer’s Disease (AD) is the most commonly occurring form of dementia, appearing predominantly in late adult life [1,2]. The disease causes progressive cognitive, behavioral, and functional impairment, taking a heavy personal and financial toll on patients, their family, and social services [3]. AD thus represents the intersection of a medical condition mostly affecting the older adults and a condition marked mostly by mental impairment, affecting many life domains. Though the neuropathological features resulting in neurodegeneration are well-recognized, primary pathogenic factors and processes are still unclear. Hence, no effective treatments have been found to prevent the onset nor progression of the disease and much attention is directed at primary prevention [4,5,6]. Primary prevention usually entails lifestyle changes in smoking, physical activity, and diet, in order to reduce abnormally high blood pressure and cholesterol, and managing major depression, overweight or obesity, and diabetes, if present [7].

These modifiable risk factors are mostly behavioral, reflecting an individual’s potential for preventing dementia [8]. As such, they are critical to assess for individuals and even more so for policy makers, to ensure a more informed position when considering public health programs. These health-related behaviors (e.g., smoking, diet, exercising) are indeed enacted at the individual level, yet they are shaped at the meso (e.g., workplace, neighborhood) and macro levels, rooted in identities linked to social groups [9]. Accordingly, health behaviors are predicated by social determinants such as age, social class, gender, ethnicity, and health system [9], and audience segmentation can be an effective strategy for identification and communication.

Health behavior theories focusing on the micro level emphasize cognitive variables such as beliefs (e.g., efficacy beliefs) and affect, addressing both conscious and unconscious processes. Attitudes, an evaluative response conceptualized as having both cognitive and affective components [10], are a focal construct in many health behavior models, and have been found to be predictive of health behaviors [11,12]. Indeed, knowledge, attitudes, and behavior constitute a continuum in health promotion efforts [13,14,15] and are also associated with social determinants [16]; for example, education is associated with higher knowledge and engagement in health promoting behaviors [17,18]. Attitudes towards AD have been targeted in public policy in order to make communities more “dementia-friendly”, and possibly engage people with dementia and their care givers in the considerations or decisions affecting their lives [19,20]. More broadly, attitudes toward disability are associated with expectations, access to public services, social inclusion and acceptance by family, friends, and healthcare professionals [21].

Knowledge, though not a focal construct in health behavior theories, is often a prerequisite for behavior. In order to engage in a recommended lifestyle and access services (social or therapeutic), people first need to know about the condition and its protective and risk factors. Knowledge about a health condition is a critical first step in facilitating appropriate and timely use of preventive health services, either by laypersons or the healthcare system; for example, older persons who know more about AD and attach less stigma to it may be more inclined to discuss early signs with their healthcare practitioner [15,22]. Evaluating knowledge and attitudes of the population can also guide psychoeducational efforts among support groups or social services staff [23,24,25].

The meanings of health, illness, and necessary care or cure are culturally constructed [26,27], i.e., they are experienced within shared beliefs (attitudes), values, and norms of a given racial or ethnic group [28,29,30]. Hence, examining AD-related attitudes and behaviors within a cultural context is pertinent [31]. This is even more crucial in the case of AD, where aging and mental health intersect. AD occurs mostly in late adulthood, and ageism is highly pervasive [32]. Moreover, the symptoms of AD are mostly cognitive and behavioral, connecting AD with a mental disorder [33], also often stigmatized [28,29], and associated with delayed help seeking [34]. However, few studies have reported on AD-related attitudes and preventive behaviors in a cross cultural context [22,35,36]. Israel, a culturally diverse society, provides a natural laboratory for such examination, and characteristics of two main population groups will be reviewed pertaining to old age and mental health stigma.

### 1.1. The Israeli Context

#### 1.1.1. Palestinian Citizens of Israel

Palestinian Citizens of Israel (PCI) are an ethnic minority that constitute 21% of the country’s population, most of them Muslims [37]. They experience social exclusion and discrimination [38,39,40], resulting in higher rates of poverty and lower educational attainment [37]. PCI differ from the Jewish majority in language, religion, and other cultural characteristics [41,42]. Some of the values characterizing this group have implications for the status of older adults; specifically, values of collectivism, loyalty to kin, interdependence, and vertical hierarchy [43] are reflected in dense social networks, mutual aid and support, and collective management of households [44]. Indeed, the majority of PCI older adults live in a two- or multi-generational housing setup [45] and patriarchal norms and customs dictate that the older adults command respect and maintain economic control [46]. Although recent effects of modernization in PCI society have fractured the previous dominant position of the older adults [43,47], scaffolding and deference to them are still the norm. Thus, endemic low educational attainment and low income are expected to be associated with less AD-related preventive behavior, compared with the Jewish majority. However, the comparative position of their attitudes towards AD is unclear. On the one hand, respect for older adults could be associated with more accepting attitudes for dementia and AD. On the other, negative stigma pertaining to mental health, and specifically social distance from individuals with mental illness among Arab populations [48] could be reflected in less accepting attitudes towards individuals with AD.

#### 1.1.2. Former Soviet Union Immigrants

A large influx of immigrants from the Former Soviet Union (FSU) entered Israel in the 1990s and they currently constitute 9% of the population [49]. FSU immigrants are older as a group and possess higher educational attainment, yet their average income is below that of the long-term Israeli Jew (LTIJ) population [50,51]. Old FSU immigrants exhibit cohort effects: they experienced major negative life events such as war, religious persecution, Communist rule, inadequate medical care, nutritional deprivation, immigration, and economic instability [52]. Studies consistently report higher psychological distress and psychiatric morbidity among FSU immigrants compared to the local population (both in the U.S. and Israel) [53], at least in the first years following immigration [54]. However, immigrants from FSU hold negative attitudes towards mental health services and thus are more likely to somaticize their complaints and channel them to primary care providers [55]. They report a negative stigma towards mental illness, which is viewed as a lack of “dusha”, a Russian word meaning inner strength and moral character [56], and individuals with mental illness are regarded with a high level of stigma [56]. Thus, FSU immigrants can be expected to have high levels of AD-related preventive behavior due to their high educational attainment, yet their attitudes regarding to AD are expected to be more avoidant and evasive.

**Figure A1:**
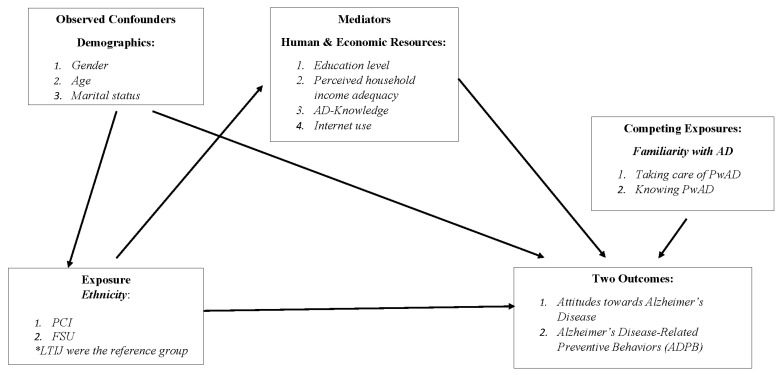
Direct acyclic graphs (DAG) of the relationship between the study variables.

In addition to the typical social determinants of health (age, gender, marital status, education, income), two additional variables need to be considered as potentially associated with AD-related attitudes and behavior. First, exposure/familiarity with AD was found to be associated with enhanced AD knowledge and more accepting attitudes [20,24], and familiarity with mental illness was found to be associated with reduced stigma [57], decreased desire for social isolation and increased positive attitudes towards people with mental health issues. Second, Internet use, which affords greater access to health information [58,59], may also be associated with AD-related attitudes and behavior. Accordingly, these variables were also included in the current analysis. Figure A1 presents the conceptual model of the study. Using direct acyclic graphs (DAG) terms [60], ethnicity (or population groups) is considered an exposure/independent variable and outcome/dependent variables are attitudes towards AD and AD-related preventive behaviors. Demographic characteristics (age, gender, marital status) are considered as confounders. Human and economic capital (income, education, Internet use and AD knowledge) are considered potential mediators. AD-familiarity is considered a competing exposure variable.

### 1.2. Research Goals, Questions and Hypotheses

The present study aimed to examine: (1) whether ethnic differences exist in attitudes towards AD and engagement in preventive behaviors, and (2) whether demographic, human and economic resources, and familiarity with AD change these group differences in one or the two outcome variables. Therefore, based on previous research, we hypothesized that:

**H1.**  *As both immigrants from the FSU and PCI share negative views of mental health, the attitudes of each group toward AD are less accepting, compared to LTIJ; as PCI culture is more respectful toward old age, PCI harbor more positive attitudes compared to FSU immigrants*.

A Research Question is posed as to whether differences in attitudes, if identified, remain after including demographic, human, and economic resources, and familiarity with AD.

**H2a.**  *Due to educational attainment, preventive behaviors are highest among FSU immigrants, followed by LTIJ and then PCI*.

**H2b.**  *These group differences decrease or disappear after including demographic, human, and economic resources, and familiarity with AD*.

## 2. Materials and Methods

### 2.1. Participants and Procedure

Data were drawn from two waves of the Israeli branch of the Survey of Health, Aging, and Retirement in Europe (SHARE-Israel), which comprises a national-representative sample of Israeli households of adults aged 50 or older and their spouses (the latter—regardless of age) [61]. The design was based on a probability sample of households within 150 representative statistical areas delineated by geographical and sociodemographic criteria. More details on SHARE-Israel can be found on its official website (http://igdc.huji.ac.il/englishsite/share/home.aspx (accessed on 11 August 2020).

Respondents were interviewed during 2015 (Wave 6, n = 2727) at which time they were asked about Internet use (n = 2025) and demographic characteristics. They also responded to a supplementary paper drop-off questionnaire focusing on AD during 2017 (n = 1638 respondents to drop-off out of 2131 respondents to Wave 7). The data were collected by means of comprehensive face-to-face interviews using computer-assisted personal interviews, each of which lasted about 90 min, and a supplementary paper drop-off questionnaire, which was returned later. Informed consent was obtained from all respondents prior to the interview. SHARE-Israel received ethical approval from the Institutional Review Board of the Hebrew University of Jerusalem and the general survey had a countries-wide institutional review board [62].

As the survey questions regarding AD were included in the drop-off questionnaire administered in Wave 7, the sample of the current study was limited to the 1198 respondents who completed this questionnaire at both Waves 6 and 7. Response to the surveys was either in Hebrew, Arabic, or Russian, the three major languages spoken in Israel among adults aged 50 and over (see Table 1).

### 2.2. Measures

Measures are described according to the DAG model (Figure A1, see Appendix A).

#### 2.2.1. Outcome Variables

**Attitudes towards Alzheimer’s Disease.** Participants were asked to indicate the extent to which they agree with two statements: “It is difficult for me to talk to someone with AD” and “I feel comfortable being around people with AD” (reversed). Response was on a five-point Likert scale, ranging from “totally disagree (=1)” to “very much agree (=5)” so that higher scores denoted negative attitudes towards persons with AD. The correlation between the two items was *r* = 0.27, *p* < 0.001. Additional attitude statements, not in the current analysis, pertained to the possibility of joy in life with AD and the value of personal life if afflicted with AD. These latter items had very low positive correlation with any other item.

**Alzheimer’s Disease-Related Preventive Behaviors (ADPB).** Participants were asked to indicate whether they had engaged in nine behaviors on a regular basis in the preceding month. The nine behaviors were: physical exercise; consumption of foods with high saturated fat and cholesterol; consumption of green vegetables, nuts, cereals, fish or olive oil; limitation of total daily caloric intake; reduction of stress level for the purpose of reducing, preventing, and/or coping with stress; communication with family or friends; participation in social activities; doing number games such as crossword puzzles or Sudoku; and taking nutritional supplements, e.g., Vitamin B12, Vitamin E, and Omega 3. The items were summed such that the score could potentially range from 0 to 9. Internal reliability of the scale was good, α = 0.62, considering that dichotomous items comprising a scale carry lower associations [63].

All items (attitudes and behavior) were pre-tested by SHARE-IL in two small samples.

#### 2.2.2. Independent/Exposure Variable

**Ethnicity.** The population groups were LTIJ, PCI, and FSU immigrants. They were identified based on language of interview and other information from the interviews (native language, residence, country of birth, immigration date), allowing SHARE Israel to create this grouping variable. Respondents were assigned to a population group and included in the analyses only if they were identified by these variables [64]. Based on these definitions we constructed two dichotomous variables: PCI and FSU immigrants. LTIJ were the reference group.

***Competing Exposure***

**Familiarity with Alzheimer’s Disease.** Participants indicated in two items whether they know/knew somebody with AD (with a list of six options such as spouse, parent, relative, etc.) and whether they take/took care of a person with AD (PwAD). The response options included: consistent care/help for someone with AD (currently or in the past), irregular care/help, or none. A response to either of the first two options was coded as ‘1’, and response to ‘none’ was coded as ‘0’. Based on these items we constructed two dichotomous variables: know/knew a person with AD and take/took care of a person with AD (coded as “1” or “0”).

#### 2.2.3. Mediators (Human and Economic Resources)

**Education level**. Response was coded by one of seven categories classified by the International Standard Classification of Education [ISCED] [65], ranging from 0 (preprimary) to 6 (second-stage tertiary education).

**Perceived household income adequacy.** Respondent were asked “Thinking of your household’s total monthly income, would you say that your household is able to make ends meet?” The answer scale was composed of four categories ranging from “with great difficulty” to “easily”.

**Knowledge of Alzheimer’s Disease.** Questions tapping general knowledge about AD were adapted from previous measures [19,22,23]. All 10 items were pre-tested in 2 prior Israeli samples by the SHARE-IL. Responses were provided on a three-point response scale: correct/incorrect/I don’t know. Sample items were “Alzheimer’s Disease could be contagious” (incorrect, [22]), “Alzheimer’s Disease can be diagnosed with a blood test” (incorrect, [22]) and “symptoms of severe depression can be mistaken for symptoms of AD“ (correct, [23]). Correct responses were summed such that the AD knowledge score ranged from 0 to 10. Internal reliability of the scale was good, α = 0.63, considering the dichotomous items comprising the scale, leading to lower associations [63].

**Internet Use.** Participants were asked whether they used the Internet in the preceding week for e-mail, information search, shopping, or any other purpose at least once. Responses were on a dichotomous Yes/No scale (coded as “1” or “0”).

#### 2.2.4. Confounders

**Demographic characteristics** included age in years, gender (1 = women; 0 = men) and marital status.

### 2.3. Data Analysis

We first performed univariate descriptive analyses, characterizing participants, and presenting the outcome variables by group (LTIJ, PCI, and FSU immigrants). Due to the large number of comparisons in the outcome variables of attitudes towards persons with AD and AD-related preventive behaviors, a Bonferroni correction was applied to reduce the type 1 error rate. This consisted of dividing the alpha level by the number of comparisons [66]. Then, for a better understanding of the differences between groups, hierarchical linear regressions were conducted. The regressions were performed in three steps. In the first step, dichotomous ethnic variables (PCI and FSU immigrants, compared to the LTIJ) and demographic variables (gender, marital status, and age) were entered. In the second step, the human and economic resources variables (education, perceived income adequacy, Internet use, and AD knowledge) were added. In the third step, familiarity with AD was added. Data were analyzed using SPSS statistical software, PC version 26.0 (IBM Corp., Armonk, NY, USA).

## 3. Results

### 3.1. Sample Characteristics

Characteristics of the participants are presented in Table 1. Panel A describes the demographic and socio-economic characteristics of the sample. Most of the sample were LTIJ (78.0%), while 10.7% were PCI, and 11.3% were immigrants from the FSU since 1989. As can be seen from Table 1, significant differences were found between groups, both in socio-demographic characteristics as well as in our dependent variables (Panel B).

In all three groups, women were the majority, especially among FSU immigrants (59.2%). Age difference was found between groups: while the mean age in the current sample at Wave 7 was 71.59 (SD = 9.25), the mean age among FSU immigrants was significantly higher (Mean = 75.44, SD = 8.98) compared to LTIJ and PCI (Mean = 70.96, SD = 9.31 and Mean = 71.22, SD = 8.09 respectively), F(2) = 33.310, *p* < 0.001, η^2^ = 0.053. The highest percentage of respondents who were living with a partner was among PCI (83.15%), followed by LTIJ (72.15%) and the lowest percentage was among FSU immigrants (63.27%).

In line with the literature, a significant difference in educational attainment was documented between groups. The highest level of education was among FSU immigrants, then LTIJ and the lowest level was among PCI (67.3%, 31.9%, and 6.3% hold academic degrees, respectively). Similarly, the highest mean score on the Knowledge of AD index (see Panel B) was among FSU immigrants (Mean = 5.22, SD = 2.13), followed by LTIJ (Mean = 4.68, SD = 2.38) and PCI (Mean = 4.16, SD = 1.87), F(2) = 43.485, *p* < 0.001, η^2^ = 0.014. However, the highest perceived income adequacy was among LTIJ, then FSU immigrants and lowest was reported among PCI (42.6%, 13.3% and 9.0%, respectively). In addition, among PCI only about 10.2% used the Internet in the seven days prior to the interview, while among FSU immigrants the percentage was 50.7% and among LTIJ, 58.7%.

LTIJ also reported the highest percentage (52.9%) of respondents who know/knew someone with AD, followed by FSU immigrants (51.8%) and PCI (47.7%). Concomitantly, LTIJ reported the highest provision of care for someone with AD on regular or infrequent basis (17.1%), followed by FSU immigrants (14.7%) and PCI (12.5%). However, these differences between groups on both items (know/knew someone with AD and take/took care of a PwAD) were nonsignificant (*χ*^2^ = 1.125, *p* = 0.533 and *χ*^2^ = 2.07, *p* = 0.355, respectively).

**Table 1:** Descriptive statistics and univariate analyses.

Panel A: Demographic and Socio-Economic Characteristics							
		LTIJ	PCI	FSU Immigrants	Total	F/*χ*^2^	Partial η^2^
	N	934	128	136	1198		
	%	78.0%	10.7%	11.3%	100%		
Gender, % women	%	57.9%	54.4%	59.1%	57.6%	46.157 *	0.001
Age	Mean	70.96	71.22	75.44	71.59	32.429 *	0.005
	SD	9.31	8.09	8.98	9.25		
Marital status, % married	%	72.1%	83.1%	63.2%	72.3%	21.414 *	0.020
Education:						161.546 *	0.214
0. Never attended an educ. program 1. Primary education 2. Lower secondary educ. 3. Upper secondary educ. 4. Postsecondary nontertiary educ. 5. Bachelor’s or equivalent level 6. Master’s or doctoral equivalent level		3.2%	21.0%	0.5%	5.0%		
		19.4%	47.7%	1.0%	20.3%		
		11.8%	14.8%	1.5%	10.8%		
		29.2%	9.7%	10.7%	24.3%		
		4.6%	0.6%	18.9%	6.0%		
		31.5%	6.3%	66.8%	33.2%		
		0.4%	0.0%	0.5%	0.3%		
Perceived income adequacy						45.947 *	0.071
1. With great difficulty 2. With some difficulty 3. Fairly easily 4. Easily		10.1%	16.4%	13.3%	11.3%		
		20.8%	47.5%	35.1%	26.0%		
		26.5%	27.1%	38.3%	28.1%		
		42.6%	9.0%	13.3%	34.6%		
Use of internet in past 7 days, yes	%	58.7%	10.2%	50.7%	52.7%	106.994 *	0.089
**Panel B: AD-Related Variables**							
		**LTIJ** **a**	**PCI** **b**	**FSU Immigrants** **c**	**Total**	**F/*χ*^2^**	**Partial** **η^2^**
Know/knew a PwAD	%	53.0%	48.1%	51.8%	52.3%	1.101	
Take/took care of a PwAD	%	17.2%	12.4%	14.4%	16.3%	2.336	
Alzheimer’s Disease Knowledge	Mean	4.68 bc	4.16 ac	5.22 ab	4.69	43.485 *	0.014
	SD	2.38	1.87	2.13	2.31		
Attitudes towards PwAD	Mean	2.98 c	2.88 c	3.62 ab	3.04	42.450 *	0.065
	SD	0.81	0.70	0.81	0.83		
Alzheimer’s Disease-related preventive behaviors	Mean	3.83 b	2.70 ac	4.05 b	3.74	85.507 *	0.033
	SD	2.15	1.50	1.91	2.10		

Note. * *p* < 0.001. Comments: a, b, c averages in a row with a different sign are significantly different from each other using the Bonferroni correction. Abbreviations: AD = Alzheimer’s Disease; PwAD—person with Alzheimer’s Disease; educ. = education.

### 3.2. Group Differences in Attitudes towards AD and AD-Related Preventive Behavior

As mentioned earlier, significant differences were found between groups concerning our two dependent variables (see Table 1). Examining attitudes (higher values denote a negative attitude toward AD) towards PwAD, FSU immigrants reported the most negative views toward AD (mean = 3.62, SD = 0.81) compared to LTIJ (mean = 2.98, SD = 0.81) and PCI (mean = 2.88, SD = 0.70), F(2) = 42.450, *p* < 0.001, η^2^ = 0.065. While the differences between FSU immigrants and each of the other two groups were significant (employing the Bonferroni correction for multiple comparisons), the difference between LTIJ and PCI was nonsignificant. Thus, our H1 regarding differences in attitudes towards AD between groups was partially supported.

Table 1 also indicates that the highest AD-related preventive behaviors score was found among FSU immigrants (mean = 4.05, SD = 1.91), followed by LTIJ (mean = 3.83, SD = 2.15) and PCI (mean = 2.70, SD = 1.50), F(2) = 85.507, *p* < 0.001, η^2^ = 0.033. However, the differences in AD-related preventive behaviors between FSU immigrants and LTIJ were nonsignificant, while the differences between PCI and the two other groups were significant, even after the Bonferroni correction. Thus, these findings also partially supported our H2a.

### 3.3. Do Group Differences Persist in Multivariate Analyses?

#### 3.3.1. Group Differences in Attitudes towards Persons with AD

As can be seen in Table 2, FSU immigrants reported the most negative attitudes towards AD (β=0.616, p<0.001) compared to both PCI and LTIJ, while controlling for demographic variables (Model 1). No significant differences between PCI and LTIJ were found (β=−0.09,p=0.226). Adding human and economic resources variables (Model 2), and familiarity with AD variables (Model 3) did not change the group differences. R^2^ (the final step) was 9.7%.

Notably, no significant associations were found between the variables added in Model 2 (education, perceived income adequacy, Internet use, AD knowledge) and attitudes towards persons with AD. However, AD familiarity (Model 3) added to the explained variance (1%), with having taken care of a PwAD being associated with less negative attitudes towards AD. Lastly, age was significantly positively associated with attitudes: older respondents held more negative attitudes toward persons with AD.

#### 3.3.2. Group Differences in AD-Related Preventive Behaviors

Table 3 presents the linear hierarchical regression models predicting AD-related preventive behaviors. While controlling for demographic variables (Model 1), the difference between LTIJ and PCI remained (β=−0.17, p<0.001), so that PCI reported lower engagement in such behaviors. No significant difference was found between LTIJ and FSU immigrants (β=0.03, p=0.338). Controlling for human and economic capital variables (Model 2), specifically Internet use and AD knowledge, significantly decreased the disadvantage of PCI (β=−0.09, p<0.001). Adding the familiarity with AD variables (Model 3) did not change the gap between LTIJ and PCI. In this context, it is important to emphasize that AD knowledge had the greatest association with AD-related preventive behavior (β=0.32,p<0.001). In addition, it was also found that Internet use was significantly positively associated with AD-related preventive behaviors. In contrast, perceived income adequacy was non-significantly associated with AD-related preventive behaviors. R^2^ (the final step) was 27.2%. Thus, H2b pertaining to decrease or disappearance of group differences in AD-related preventive behaviors was supported among PCI: group differences in AD-related preventive behaviors decreased after including demographic characteristics, human and economic resources variables, and familiarity with AD variables.

## 4. Discussion

The current research focused on group differences in attitudes towards AD and the uptake of AD-related preventive behaviors among adults in middle and late adulthood affiliated with different ethnic groups in Israel. Despite growing interest and research in the neuropsychology of dementia and the primary prevention of AD [4,5], ethnic differences in the context of AD have received relatively little attention. This research aimed to fill this void.

The main finding emerging from our study was the group differences in attitudes toward AD. FSU immigrants reported significantly more avoidant and evasive attitudes toward PwAD than the other two groups, while PCI respondents held the most positive attitudes; this latter difference was significant in comparison to FSU, although not in comparison to LTIJ. Taking into account human and economic resources and AD familiarity did not eliminate this difference. The second main finding was the group differences in ADPB, where PCI respondents reported the lowest engagement in preventive behaviors, significantly less than the other two groups. This gap decreased significantly after controlling for both AD knowledge and Internet use.

The difference in attitudes—not eliminated by the various mediating and competing exposure variables—may indicate that these are deep-rooted differences, unaffected by between-group socio-economic attributes and emanating from cultural background. Although a recent review of attitudes towards people with disabilities revealed that in many samples people hold less positive attitudes towards intellectual disability [21], this review did not address culture and found conflicting evidence regarding religious beliefs. The interpretation that attitudes towards illness, especially mental illness, are culturally constructed, is supported by previous literature [67,68]. The relatively negative attitude towards PwAD exhibited by immigrants from the FSU may be construed as reflecting attitudes towards mental illness. Basic assumptions in the Soviet construal of persons with mental illness are the existence of a flaw and a lack of “dusha” [67,69]. In the Soviet society, which valued people by their contribution to the workforce, individuals with mental illness were regarded as non-beneficial, unnecessary, and perhaps even “redundant”.

However, despite the shared negative views of mental health both by immigrants from the FSU and by PCI [48,70], the attitudes of the latter toward PwAD were different. One possible explanation is the greater respect inherent in Arab culture toward old age and the older adults, resulting in more positive attitudes toward PwAD than among either FSU immigrants or LTIJ (though not significantly different from the latter group). Another possible explanation is that the relative material and political disadvantages experienced by the PCI group may foster prosocial attitudes and behaviors [71,72], contributing to greater community solidarity, exhibited here in the context of AD.

The findings on AD-related preventive behaviors, namely that PCI reported less uptake and that these differences decreased once human and economic resources were accounted for, is consistent with findings in other samples. Indeed, PCI generally engage in fewer health behaviors (e.g., less physical activity and more smoking, though conversely more fruit and vegetable consumption) than LTIJ and this difference is attributed to social economic status [73,74], specifically education and income [74].

Though AD knowledge was not a dependent variable in this study, interesting findings on its dispersion among the groups and the amendment of group differences emerged. AD knowledge was highest among immigrants from FSU, followed by LTIJ and lowest among PCI. These findings are in line with the research literature documenting ethnic differences in human capital among these groups in Israel [51] and can be attributed to the educational attainments of people in these three groups. Moreover, knowledge was not associated with attitudes towards PwAD but, along with Internet use, was associated with AD-related preventive behaviors, so that their inclusion in predicting uptake of behaviors decreased the group difference. This illustrates the capital enhancing effect of knowledge and of using the Internet, transforming information acquisition into health-related behaviors. It should be noted that informal knowledge, gained from exposure to PwAD, was also positively associated with attitudes towards PwAD and AD-related preventive behaviors, echoing previous findings on the association between exposure (in various indicators) and attitudes towards persons with disabilities [21] or mental illness (e.g., [57,75,76]).

These findings add to the growing body of research literature indicating that an increase in knowledge, including of risk factors (e.g., the LIBRA, [5]), is a pivotal first step for modifying individuals’ health behaviors and lifestyle patterns [77]. Knowledge is malleable: health education efforts focused on addressing the ethnic gap in AD knowledge could later impact behavior. The strong association between AD knowledge and AD-related preventive behavior has important implications for promoting ethnic health-related equity. The findings about the differences in attitudes highlight the importance of culturally tailoring content, so that better AD knowledge can be provided to designated communities. Our results indicate that educational efforts should focus on closing this gap in AD knowledge for the PCI population while seeking ways to modify the negative stigma among FSU immigrants. This latter goal is plausible: even though values or beliefs do not change quickly [78], there is slight evidence that attitudes regarding mental health could change among this group [56].

Thus, we believe that public education about AD that takes cultural attitudes into account can increase public acceptance of people with AD [79], promote beliefs that dementia risk reduction is possible, and increase motivation for behavioral changes [80]. This, in turn, may hopefully lead to increased inclusion as well as better preventive behaviors among all citizens in the different ethnic groups, better identification of symptoms, and greater seeking of services [24]. Notably, interventions should go beyond information transmission and drawing attention to personal risk and prevention. Rather, they should include attitudes and beliefs, incorporate preliminary work gauging perspectives of healthcare providers from different professions, carers, local stakeholders (local municipalities, community centers, companies), and “regular” community members, all disaggregated into the salient ethnic or cultural groups in societies. This will allow to prepare and disseminate segmented materials both for the public-at-large, stakeholders, and for healthcare providers, among the latter in the training stage (tertiary and non-academic post-secondary) and in continuing education. The healthcare providers audience could receive direct instruction, but also simulations and case studies on ethnic differences pertaining to AD in order to enhance their cultural awareness, knowledge, and competence. The above directions can be incorporated into the in-planning Israeli National Program for aging.

Lastly, in line with previous literature [81,82], we found that Internet use was associated with an uptake of preventive behaviors. However, due to the limitations of the SHARE database, we could not investigate which specific digital activities are associated with enhanced uptake of preventive behaviors in our sample. We thus recommend developing specific items on digital engagement in further surveys.

This study has a number of strengths. First, the representativeness of the sample and the large number of respondents (n = 1198) allows generalization of the findings to the Israeli context, even though the sample was representative of households and not of individuals and not all respondents returned the drop-off questionnaire. Second, the analyses employed in the study make it possible to unravel whether and which characteristics decrease group differences in attitudes and behaviors, thus paving the way for future interventions. Third, similar analyses could be carried out in other countries, based on group composition (ethnic, racial, regional). However, certain limitations of the study should be noted. Some were imposed by the SHARE database. The SHARE survey does not facilitate the examination of group differences in preventive behaviors or other variables in other immigrant groups in Israel, significantly smaller than those from the FSU, such as immigrants from Ethiopia or Western immigrants. Second, all of the variables were self-reported by the respondents and came from a single source. Some variables (e.g., demographics) are inherently reliable and valid, some are reported practices (e.g., physical activity) which are more prone to self-presentation bias, and some are inherently perceptive (attitudes towards a PwAD). Third, our dependent variables carry some methodological weakness. The AD-related preventive behaviors variable exhibited relatively low reliability, most likely as it was based on dichotomous responses [63] and covered different domains (e.g., behaviors pertaining to diet, exercise, and social interaction). Similarly, the two items composing the attitude variable had a low, yet significant, association between them.

## 5. Conclusions

The results of this study confirmed that group ethnic differences appear among middle and older adults both in attitudes towards PwAD and uptake of AD-related preventive behaviors. An examination of these ethnic differences is especially crucial in the case of AD, where aging and mental health compound to form negative stigma. An understanding of these ethnic differences may help to tailor culturally appropriate psychoeducational interventions and effective services for PwAD and their care givers [23,24,25].

## Figures and Tables

**Table 2:** Hierarchical linear regression—group differences in attitudes towards persons with Alzheimer’s disease.

	Model 1			Model 2			Model 3		
	B	SE B	β	B	SE B	β	B	SE B	β
Constant	2.47 ***	0.22		2.62 ***	0.24		2.65 ***	0.24	
Ethnicity (LTIJ served as a reference group, 0)									
PCI	−0.09	0.08	−0.03	−0.13	0.08	−0.05	−0.14	0.08	−0.05
FSU immigrants	0.62 ***	0.08	0.23	0.65 ***	0.08	0.25	0.64 ***	0.08	0.24
Gender (men = 0)	−0.07	0.05	−0.04	−0.06	0.05	−0.04	−0.05	0.05	−0.03
Age	0.01 ***	0.00	0.09	0.01 ***	0.00	0.09	0.01 ***	0.00	0.09
Marital Status	−0.07	0.06	−0.04	−0.06	0.06	−0.03	−0.05	0.06	−0.03
Education				−0.02	0.02	−0.03	−0.02	0.02	−0.04
Perceived household income adequacy				−0.01	0.03	−0.01	−0.02	0.03	−0.02
Internet use				0.04	0.06	0.02	0.05	0.06	0.03
AD Knowledge				−0.02	0.10	−0.05	−0.01	0.01	−0.03
Taking care of a PwAD							−0.18 **	0.07	−0.08
Knowing a PwAD							−0.08	0.05	−0.05
R^2^	0.083			0.087			0.097		
R^2^ change				0.004			0.010		

Note. *n* of models = 1198. *** *p* < 0.001; ** *p* < 0.01. R^2^ change refers to added explained variance between the steps. Abbreviations: PwAD = person with Alzheimer’s Disease; PCI = Palestinian Citizens of Israel; FSU = Former Soviet Union.

**Table 3:** Hierarchical linear regression—group differences in Alzheimer’s disease-related preventive behavior.

	Model 1			Model 2			Model 3		
	B	SE B	β	B	SE B	β	B	SE B	β
Constant	3.93 ***	0.56		1.198 ***	0.55		1.16 *	0.53	
Ethnicity (LTIJ served as a reference group, 0)									
PCI	−1.13 ***	0.19	−0.17	−0.59 ***	0.19	−0.09	−0.55 ***	0.19	−0.08
FSU immigrants	0.33	0.19	0.05	0.14	0.19	0.02	0.13	0.18	0.02
Gender (men = 0)	0.23	0.12	0.05	0.27 *	0.12	0.06	0.16	0.11	0.04
Age	−0.01	0.01	−0.03	0.00	0.01	0.03	0.00	0.01	0.02
Marital Status	0.25	0.14	0.05	0.10	0.13	0.02	0.08	0.13	0.02
Education				0.01	0.04	0.01	0.03	0.04	0.02
Perceived household income adequacy				−0.02	0.06	−0.00	0.02	0.06	0.01
Internet use				0.69 ***	0.13	0.17	0.58 ***	0.13	0.14
AD Knowledge				0.34 ***	0.02	0.38	0.29 ***	0.02	0.32
Taking care of a PwAD							0.91 ***	0.15	0.16
Know/knew a PwAD							0.46 ***	0.12	0.11
*R* ^2^	0.037			0.226			0.272		
*R*^2^ change				0.188			0.046		

Note. *n* of models = 1198. *** *p* < 0.001; * *p* < 0.05.

## Data Availability

The datasets analyzed during the current study are available both in the SHARE website and upon request from the corresponding author.
